# Aging with my family: a grounded theory approach on the role of family when aging as foreign-born

**DOI:** 10.1186/s12877-023-04641-3

**Published:** 2024-01-10

**Authors:** Anna Zhou, Ingrid Hellström, Susanne Roos, Åsa Larsson Ranada

**Affiliations:** 1 Department for health, medicine and caring sciences, HMV, Linköping University, Linköping, Sweden; 2 Department of Health Care Sciences, Marie Cederschiöld University, Linköping, Sweden

**Keywords:** Family relationships, Older migrants, Community, Family solidarity

## Abstract

**Background:**

Research indicates that it is the quality of the closest relationships in the mixture of social relations that matters most for older adults. For older foreign-born, especially those who migrate late in life, the family is often the only socioeconomical resource they can lean on. This study aims to explore how older foreign-born perceive the role of family as they age.

**Methods:**

The study design has a grounded theory approach. Data consist of individual open-ended interviews with 15 foreign-born informants aged between 60 and 85 years old who migrated to Sweden as adults from various parts of the world.

**Results:**

The findings demonstrate that family was an essential part of the informants’ lives as they lived for their families and their families lived for them. Family solidarity was described as a cultural heritage they took over from their original families and a cultural heritage they wished to pass on to their future generations. They found that this was what separated them as foreign-born from native-born. Memories of their parents reminded them of their biological, social, and cultural heritages. The intimate relationship with their spouses in a life course had served as a source of validation of their individual identities and promoted personal growth and self-esteem. The role as a loving and caring parent entailed a sense of accomplishment and satisfaction for the life lived. And now as grandparents, the role as a link between the family’s historical heritage and the future generation entailed not only a sense of coherence as they aged but also hope and meaning beyond their own lives.

**Conclusions:**

The older foreign-born experienced life satisfaction as they aged with their families. Family meant community and solidarity. It was in the family that they found their distinct roles that had defined them. Family was an indispensable part of their social identity. The findings highlight the importance of older foreign-born being studied from a family and lifetime perspective.

## Introduction

Most of us live our entire lives in the context of a family. Family is probably the most enduring and consequential social relationship for many during our life. Two concepts employed in anthropology and sociology in discussing the family are structure and function [1, 2]. Structure is about who belongs to the family. In most industrialized Western countries, Sweden included, the nuclear family with the parents and the children, appears to predominate the type of family system. In the rest of the world, however, extended family with at least three generations with the grandparents, the parents, the children, and even other kin are usually considered to be the family. The functions refer to how the family satisfy its members´ needs to survive as a social unit. It´s generally agreed that the family has four universal basic social functions: common residence, economic cooperation, reproduction, and socialization/education [1, 2]. While the structure of family varies a great deal both across cultures but also historically in individual countries along with other aspects of social developments – nuclear families are increasing in all countries in the world and even single-parent families and homosexual families are becoming more common today, the basic universal functions of the family being a physical and emotional support to its members remain unchanged strong across all societies in the world [1–3].

Throughout our lives we exchange instrumental, emotional, informational, as well as financial support within the family. The family may become even more important as we age. For example, the socioemotional selectivity theory [4] argues that as people age and their realization of the finitude of life is reinforced with time, they tend to restrict their networks and concentrate their affective energies on those people who are most important to them. Research indicates that marital relationships and intergenerational support in adult child-parent relationships, being a source of either stress or support, play a central role in shaping older people’s psychological well-being [5]. Research in the field indicates that migration is rarely based solely on individual decisions but is usually based on a family strategy in search of a better life for the family as a whole [6–8]. Research has also found that family responsibilities and family obligations persist regardless of distance in time and space [9, 10]. For older foreign-born people, especially those who migrate late in life, the family is often the only socioeconomical resource they can lean on, and supportive family relationships are crucial for these older people’s well-being especially as it relates to loneliness and social isolation [11]. Studies of non-European migrant groups in England and Wales indicate that family remains central in the provision of care and support for these ethnic minority older people [12, 13]. Even in Sweden, feelings of family obligation and norms of filial piety are strong among families with Middle East origins [14, 15], and family decisions affect older foreign-born people’s use of public service [16–18].

Older foreign-born is a rapidly growing population in Sweden. At the end of 2021, about 14% of the foreign-born population were 65 years and older, compared to 6.3 per cent in 2000 [19]. The older foreign-born population in Sweden is a diverse group with a wide variation of backgrounds. They came from various parts of the world. They migrated to Sweden at various stages of their lives and for varied reasons. Most of those who arrived before the 1980s were young workers from other European countries and have had a long working life in Sweden. During the last two decades, however, there came many non-European migrants to Sweden for asylum or family reunion. That is, the demographic composition and structure of foreign-born population is also changing [20].

The living situations of these older foreign-born people have often been labelled as *double jeopardy* [21] or *twice poor* [22] by researchers who argue that the intersectionality of older age and lack of social, economic, and cultural resources make these older people, especially those who migrated to Sweden late in life, a particularly vulnerable group and a potential challenge for the Swedish community healthcare initiatives [18, 22–24]. Previous studies indicate that the older foreign-born in Sweden are socioeconomically less resourceful compared to natives of the same age [22], an observation also noted in international research [25]. However, there is still little knowledge about how these older foreign-born in Sweden experience their overall living conditions [26]. The role of family for older foreign-born has been studied only in the context of care provision [17, 18]. All these studies were conducted from the perspective of care managers who had met the older people together with their families and assessed their needs for social support, or in a few cases that of nurses who worked at residential care for older people. To our knowledge, no study has been conducted from older foreign-born people’s perspectives on how they experience their family lives and what family means for them as they age, which should be of interest not only for making these older people’s voices heard but also for supplementing a skewed research field – i.e., the knowledge gaps the present study intends to fill. Understanding these older people’s perceptions and the significance of family relationships would contribute to our knowledge about these older people’s social resources as well as their social identities. It may also shed new light on older people’s roles in the family and in society at large.

### Aim

This study explores perceptions and significance of the family among foreign-born older people living in Sweden.

## Methods

The research design is based on the Glaserian paradigm of grounded theory [27]. The sampling was purposive [28] and aimed to represent a mixture of demographic, migration status, socioeconomical, and cultural backgrounds of the target population. Country of origin was used as a proxy for cultural background. Our goal was to find informants whose origins were representative of the World Culture Map based on World Values Survey (2005–2022) [29], and in the meantime representative of the older foreign-born population living in Sweden. Demographic backgrounds consisted of age, gender, civil status, mother tongue, number of children living in Sweden and abroad. Migration status included age at the time of migration and years lived in Sweden. Socioeconomic backgrounds consisted of self-assessed health status, state of employment, and years of formal education. We tried to achieve a balanced mixture of all these factors in our sampling. However, we prioritized cultural background, migration status and age, because we believed these factors being most significant for perceptions on and experiences of aging as foreign-born.

Eligibility criteria were all foreign-born individuals 60 years or older living in Sweden and capable of expressing themselves. The first three informants were recruited by convenience sampling [28]. Then, in order to recruit informants with a variation of backgrounds, we visited places where the local older foreign-born people usually gathered, like for example ethnic cultural associations and religious communities. Representatives of these organizations were contacted to have their permission to put up announcement of our study in their locations, and to ask for their recommendation for informants that might be eligible for our study. Three informants were recruited by means of network sampling [28] in the form of recommendations by earlier informants. All informants provided written informed consent.

All 15 informants were between 60- and 85-years-old living in south-eastern Sweden. The youngest age at the time of immigration to Sweden was 17 and the oldest was 58. Six informants migrated to Sweden to be with their family, four moved to Sweden for employment, and five migrated to Sweden for asylum. The longest length of residence in Sweden was 67 years and the shortest was 20 years. Eleven were married and lived with their spouses, one was a widow, and three were divorced singles. Educational background varied between almost none and 20 years of formal education. The original home countries of these informants are scattered all over the world cultural map [25]: Asia (India, China, and Turkey), Europe (Finland, United Kingdom, Italy, Romania, and Croatia), Central and South America (Guatemala, Peru, Chile, and Bolivia), and Africa (Democratic Republic of the Congo) (see Table 1 for background information of the informants).

Data consisted of 15 semi-structured individual interviews with general and open questions about aging, including the researcher’s observation of the living environment where the interviews took place and descriptions of videos, photographs, medication lists, and hospital appointments that the informants shared with the first author during the interviews. Interview length was between 45 and 75 min, on average about one hour. The interview guide covered the following areas: health, aging, social relationships and social activities, and identity. In the end of the interview guide was also a structured questionnaire about their personal data: name, age, country of birth, years lived in Sweden, age at the time of immigration to Sweden, motive for migration, marital status and living arrangement, number of children living in Sweden and abroad, years of formal education, employment status, self-assessed health SAH (“How is your health in general?”), which has five possible answers, ranging from “Excellent” to “Very bad.” [30].

**Table 1:** Background information of the informants

No.	Age/Gender (F-woman; M-man) /Marital status	Number of children/ living arrangement	Country of origin	Age at the time of migration	Years lived in Sweden	Self-assessed health	Years of formal education	Employment status
1	68/F/widow	2/alone	India	38	30	Excellent	19	Working
2	75/M/divorced	1/alone	China	50	25	Good	16	Pensioner
3	74/F/married	4/with husband	Guatemala	38	36	Excellent	12	Pensioner
4	64/M/married	4/with wife	Turkey	19	45	Good	12	Working
5	85/M/married	2/with wife	Finland	18	67	Good	0	Pensioner
6	63/M/married	2/with wife	UK	27	36	Excellent	17	Working
7	63/F/married	3/with husband	Kongo	30	33	Good	12	Working
8	69/M/married	2/with wife	Italy	33	36	Good	12	Working
9	78/F/divorced	1/alone	Peru	56	22	Good	9	Pensioner
10	78/M/divorced	1/with daughter	Peru	58	20	Bad	9	Pensioner
11	63/M/married	2/with wife	Chile	30	33	Good	12	Pensioner
12	65/F/married	1/with husband	Finland	17	48	Good	12	Pensioner
13	60/F/married	3/with husband	Romania	23	37	Good	19	Working
14	60/M/married	2/with wife	Yugoslavia	33	27	Good	19	Working
15	71/M/married	1/with wife	Bolivia	31	40	Bad	16	Pensioner

Fourteen interviews were conducted face-to-face: eight at the informants’ homes and six in public locations. One interview was conducted via Zoom video, at the request of the informant as a precautionary measure against the risk of Covid-19 infection. Twelve interviews were conducted in Swedish, one in Chinese (the first author’s first language), and two in Spanish (a relative served as an interpreter). All interviews were audio recorded and transcribed verbatim in Swedish. A research memo was initiated shortly after each interview and field notes were made to document the research observations of the informant and the environment during the interview. Primary impressions and ideas from the interviews were also registered in the research memo. After each interview, the generated data, including analysis of the interview transcription and research memos, were discussed in the whole research team. In order to guarantee the trustworthiness of the findings we used peer-debriefing sessions with the first author who was the primary coder [31]. In addition, we also looked for disconfirming evidence [32] to ensure that the findings hereby presented do justice to both individual understandings as well as the couple’s perspectives on the issues at hand.

Data generated from the interviews together with the research memo were initially analyzed line-by-line open coding of all data pertaining to the informants´ perception on aging and their experiences of aging in Sweden. Data were constantly compared with other data, conceptualized, and coded into categories. Initial codes included, for example, “having a history/memory of my original family”, “migration as a lifetime event”, “having a shoulder to lean on/always together”, “having a role/as a link between generations” and “zooming out to our big family”. After eight interviews, we made an additional ethical application for the use of interpreter because we found it extremely important to reach “later-in-life migrants”, a group often considered to be “hard to reach” for researchers because of intersectionality of severe illness, social isolation, and other socioeconomic difficulties (as in the case of informant No. 10). We found even experiences from these informants fit into our existing categories. As the study progressed, the core category “aging with my family” emerged. We deleted the category “migration as a lifetime event” because we found migration as a lifetime event had colored their whole life and existed constantly in the background of their perception on the past, the present and the future. We also renamed the remaining categories to the present “living with the heritage of my original family”, “living with my life-mate”, “living with my children and grandchildren”, and “living with my extended family”, in order to illustrate more straightforwardly their relationships with the core category in a lifetime perspective. Then we used theoretical sampling to recruit informants who provided insight into experience of aging in Sweden as foreign-born, persons having lived in Sweden longer time and persons who had a good command of the Swedish language to express themselves freely, because we learned from earlier interviews how important language proficiency could be for how deep a person was willing to go in the conversation. A new research memo, based on the individual memos from each interview, was executed to summarize and analyze the relationship between all the categories that emerged. When the framework of emerged hypotheses was stabilized, we started to search and study the existing literature of related fields such as aging theories, migration, and family relationships. By employing emergent fit [33], the components of pre-existing theories that fit the data were then adopted as new data and compared with the hypotheses that emerged. This was done before the final conclusions were integrated.

### Ethics approval and consent to participate

The study was approved by the Regional Ethical Review Authority in Linköping, Sweden (Dnr: 2020–05672 and 2021 − 00955). Numbers have been used to protect the participants’ anonymity. Written informed consent from participants was obtained prior to interviews.

### Findings

The core category of the findings is *aging with my family*. This insight of aging entailed a sense of urgency among the informants. Life became more conscious and time more precious. They realized that life was not infinite, and they must make the best of it by spending time with people who had meant the most to them – their family members. The findings demonstrate that family was an essential part of the informants’ lives. Memories of their parents reminded them of their biological, social, and cultural heritages from their families and countries of origin. It was in the family that they found their basic needs met as human beings – the need for care, nurture, and security, the need for social community and a sense of belonging, and the need for self-esteem and esteem from others. Altogether, these interactions would have promoted their abilities to become the best versions of themselves. It was within the family that important values were promoted, upheld, and passed on. It was also within the family their identities formed and developed. It was in the family that they found the roles that defined them. The intimate relationship with their spouses in a life course might have served as a source of validation of their individual identities and promoted personal growth and self-esteem. The role as a loving and caring parent entailed a sense of accomplishment and satisfaction. The role as a link between the family’s historical heritage and the future generation entailed not only a sense of coherence as they aged but also hope and meaning beyond their own lives. The informants had a broad view of family. For them family meant above all community and belongingness, a reason why they would like to see the whole society living as one big family. The finding fits into the following four categories: *living with the heritage of my original family*, *living with my life-mate*, *living with my children and grandchildren*, and *living among my extended family*.

### Living with the heritage of my original family

The topic of aging evoked memories among the informants of their parents and their families of origins. Most reflected on their families of origins with affection. Most still had siblings, relatives, and good friends in their home countries who they talked to daily and visited regularly. However, visits to their original families were becoming less frequent after their parents had passed away, and their siblings and relatives were also getting older, or some had migrated to other places in the world. Several commented that “there was less and less to return to” their original countries.

Some informants described that their insight into aging emerged when their own parents became sick and disabled, cognitively impaired, or passed away. It was as if those memories of their parents mirrored their own future as aging and death became more realistic and palpable. Others reflected on how their parents lived and aged with their families, which gave a hint on what the rest of their own lives would look like, which aspired them to reason what the aging life was about and how to best spend their remaining days. Then they concluded that aging was all about health as everything would now stand and fall with health.

Many claimed that the most important thing for their aging lives was that family love should always live on no matter how far away they were and how long ago they left their home of origin. They would be happy enough if only they could spend time together with their loved ones, all the time, as long as they lived. They described this as a cultural heritage of family solidarity they took over from their original families and a cultural heritage they wished to pass on to future generations. They found that this was what separated them as foreign-born from native Swedes. Apart from family solidarity, there were a few other values from their original families that they still held important as life principles. One was gratitude and reverence for older people. Another was the belief in hard work and education. The third was the love for community. They regarded these as their winning recipes for coping with life. Several informants were born into Catholic families, and they had remained active members of the church, not for religious reasons but rather for its social teaching for living in the community, which they still found relevant. Migrating to a new country with different cultural practices at adult age meant they had to start over in many ways. They were, however, happy that their own children had been inspired by their hard work.

*“I think we are incredibly lucky, because we have a fantastic culture and history behind us, which came with us, and we have values, that we have taken with us, that we pass on to our children. We´ve taught them respect. We´ve taught them love. We´ve taught them hard work. We taught them to fight hard, as we ourselves had to do. You and I have had to fight to get into society. And we have transferred that. I think they have seen us and followed our examples.” (No. 1)*.

Childhood and upbringing in the original family influenced and shaped their lives in every way. While most informants had fond memories of childhood, a few had bitter memories of their loveless original families. Even in these cases, the trauma of not feeling loved and valued as a child had put them on a mission to seek a sense of purpose. One man could not hold back his tears when he described his childhood in Finland. The extreme poverty and an alcoholic and abusive father were the very reason that he migrated to Sweden in search for a better life. One woman from Romania explained that as a little girl she was regarded as less valuable than her brothers and that was why she was so happy that in Sweden she had the opportunities to study and pursue her dreams freely.

### Living with my life-mate

Seven informants migrated to Sweden to reunite with family. For them, it was more about living together with their loved ones than moving to another country. Several described that it was their devotion to their loved ones that had motivated them to learn the language and adapt to the cold climate and peculiar Swedish foods. Many had lived with their spouses since their 20s when they were married; some had even celebrated their gold and diamond wedding anniversaries. When asked how they spent their days and what they planned for aging, they answered exclusively with “we” rather than “me.” They were always together, doing everything together every day, and sharing life’s difficulties. They took long walks to the forest picking berries and mushrooms together. They went to the gym together to keep fit. They planned their retirement and household finances together. They had common friends that they socialized with together. They traveled to visit their children and their original families together. And they would stay together until one of them someday had to move to a nursing home. Although most informants had close relationships with their adult children and grandchildren, it was obvious that only their spouses were their “own” families that they were sharing their everyday lives with. They shared a whole life history and would continue to do so to the end of their lives. In one sense, they had only each other as they entered old age. They described their spouses as their best friends, life-mates, and a part of their own bodies and souls. There was much adoration and tenderness when they talked about their spouses. Several called their spouses “daddy” and “mommy” when they addressed each other – a demonstration of a long and happy marriage where the couples have grown up together and become one.

*“And she is my wife. I like her and she likes me. (laughter) We are like made for each other. Imagine there is someone smiling when you come home! Oh, so lovely!” (No. 5)*.

Several admitted they had had ups and downs in their marriages, but this was life. Their long-term marriages in old age meant a lot for these informants. It meant they always had someone to talk to and someone to share with no matter how hard life could be. After a half century together, their marriages were by far the longest intimate relationships in their lives. As one man reflected, his wife was the person who knew him best, better than his mother, because he had lived with his wife twice so long as he had lived with his mother. There were many dimensions of relationships in their long-term marriages. They grew next to each other, and they grew into each other. They strengthened each other. They complemented each other. They were not only lovers, but they were also each other’s best friends, co-workers, business partners for their households, and each other’s advisor and psychotherapist. However, this close community may also induce a sense of vulnerability because they were so dependent on each other, as one woman described her bond with her husband:

“I don’t know how it could be if I was alone. How could I take it if something happens to my children?” (No. 3).

Four of the informants were single, either divorced (No. 2, 9, 10) or widowed (No. 1). Two of them, both women (No. 1 and No. 9), were very contented with their lives as single because they had their children and grandchildren, close friends, and a broad social network. One man (No. 10) was still sad about his recent divorce after 50 years of marriage but very happy that he was very close to his daughter and her son and her husband. Only one man (No.2) did not want to talk about his own family. He argued that common values would be more important for him than blood ties, and the most important relationship would be the relationship between an individual and society.

### Living along with my children and grandchildren

All informants but one had adult children living in Sweden. Most of their children had grown up in Sweden, some born in Sweden. Several informants stated that they decided to migrate to Sweden for a better life not only for themselves but also for their children. Now after so many years, all the informants perceived Sweden as their home, the place where they would live and die, because Sweden was the place where their children and grandchildren had grown up and settled.

Many informants said they were immensely proud to be the parents to their children. They had prioritized their children’s future as their own lifetime course in Sweden. After all the years of hard work and many sacrifices, they felt their mission completed, and now it was time to relax and enjoy their children’s professional success. The distinctive joys and challenges of parenting, especially in a culture different from what they themselves had grown up in and been socialized in, had been part of a fulfilling life. Many used the expression “it’s going well for the kids” to illustrate their satisfaction with what they themselves had accomplished.

Most informants had, to their delight, very close relations with their children and grandchildren. Many said their children called them every day to check on how they were doing. They found themselves respected and still needed in the family. In the meantime, they felt nostalgic about the good old days when all the loved ones were gathered, and a frustration that the Swedish way of living with nuclear families made it difficult for generations to meet often and spend time together. One man commented his relationship with his children and grandchildren like this:

*“Oh yes, absolutely! They (authors ‘note: his children) call every day. It is the most important thing, for all foreign-born, that the culture should continue, that you always belong to the family! They should continue as I and my family in Chile. We do not see each other but we talk all the time, even though I have been away from them for 33 years. That is how I think. That is how Chileans think …… I am a grandfather but it´s just a name on paper. I never play with my grandchildren because they are not here. They rarely come. Last week my oldest grandson turned 9. They were here but the only thing he wanted to do with me was to go to the town to buy a present!” (No. 12)*.

They were still supporting their adult children actively – as problem-solvers, mental coaches, and moral supports – but they did not think their children were obliged to take care of them. They did not want to burden their children with their own problems. They would be happy enough if their children would think of their older parents and call and visit often. Only one informant was living together with his daughter and her family and depended on the municipal home care. The others were fit enough to manage on their own. They would try to keep themselves healthy and take care of themselves and live in their own houses as long as possible. What they expected from their children was more about affection than instrumental support.

Spending time with their loved ones and passing on the legacy to the next generation were described as the purpose for their remaining life. Several informants said the only thing missing in their lives now was grandchildren. Most informants already had grandchildren and had good connections with them. However, they were still looking forward to seeing them more often, being with them, doing things together with them, and hopefully developing their relationship into intergenerational friendship and solidarity. Friendship requires making time and spending time together. They wanted to demonstrate for their grandchildren sound values, perceptions, skills, and habits. They wanted to teach them things that they themselves once were good at, or they loved to do when they were young. They wanted to give their grandchildren the best they had to offer and, in the meantime, give them a chance to know their grandparents –who they were and what they had experienced. One man had already a plan for his future grandchildren:

*“I will teach them skiing because I competed in slalom when I was in Yugoslavia. I will teach them diving because I am a great diver. I will teach them skating, and fishing, because that´s something I can do. Yes, to give them time, to give them love, because the time you spend with them is love. So that they would remember me for good things. So, I am waiting to give them the best I have.” (No. 14)*.

### Living among my extended family

The informants were happy that they had inherited a love for community life from their original cultures, and they felt some discomfort to find Sweden such an individualistic society where people mostly looked after themselves and their immediate families. They argued that life was vulnerable and that individuals were not strong when they stood alone. People are all interconnected and interdependent. Many of the informants said they had good social network here in Sweden outside their families, including friends from work, through hobbies, or from faith communities or leisure associations, friends of different origins, including native Swedish and diaspora from their original countries. Some had also stayed connected with a few close friends from childhood in their countries of origin. They claimed that even though one’s social network may shrink with age, close friendship had a role of their own that was invaluable, as there were certain topics one would only want to discuss with one’s close friends. The three informants who migrated to Sweden in their 50s described their social life as mostly restricted within their families and their compatriots because of language barriers, but they thought it was good enough.

Several informants claimed that they had found meaning and purpose for the rest of their lives in doing something for others. Some worked for a charity at church or voluntary aid organizations, one worked as politician in his spare time, and others helped their older neighbors with grocery shopping. One man said he would now dedicate the rest of his life to society by sharing his 40 years of experience of successful entrepreneurship, because now it was his turn to give back. Another man, who had worked with traditional Chinese medicine all his life, was still dreaming that Swedes would learn to appreciate this alternative treatment so that he would be able to share with them his knowledge and feel himself useful in his older days. Doing something useful for society, he said, was his only concern for the future.

*“Yes, that´s the best way, if a person is to have a relationship with the society. It is the best relationship because I can make use of my last energy, and at the same time I can have exchanges with other people. Then I won´t feel lonely. I would get recognition. Then I won´t feel that my life on earth less worthy, since I have talents!” (No. 2)*.

Doing something useful for others was a way to give back, a way to express one’s gratitude. Gratitude was a word that frequently occurred when the informants talked about Sweden. Sweden was the host country that had provided them with a sanctuary of peace and prosperity. Now it was time for them to give back. Doing good for Sweden and the Swedish people would give them a sense of participation and belonging in their new homeland. They also liked to speak in terms of gratitude when talking about the role of the older person in the family and in society. They thought it was only humane to feel gratitude and reverence towards older people, who had built the society we lived in. Who we could be and what we could do could not be generated in isolation. That is, our lives depend on previous generations and on those who took care of us. One man reflected on how older people had been treated under the Covid-19 pandemic in Sweden:

*“Older people are not valued. It is the older people who have built this society. In our culture, the whole family helps to take care of the older people, as long as they live. We know that our older mother and father have toiled for us as long as they have been able to. Now when they do need help, we don´t turn our backs to them. That´s not good. I often think that Sweden turns its back on the older people. One does not forget one´s precious older parents.” (No. 4)*.

## Discussion

The informants experienced life satisfaction as they aged with their families. Their old marriages were characterized by affectionate interactions and feelings of deep affinity. They found their life´s work worthwhile when they could watch their children and grandchildren flourishing. The family was not only the source of reliable instrumental and emotional support that may have compensated for their losses due to old age and migration, but also the source of their sense of meaning and purpose with their aging lives. This is consistent with previous research showing that relationships with spouses and family members usually remain stable throughout the lifespan in the innermost circle of the social convoy of relations, and it is the quality of closest relationships that matter most to older people [34, 35].

The findings illustrated the importance of memories of the original families for older people´s experience of aging. The insight of aging reminded them that it was time for reflection on the life lived. The theory of gerotranscendence argues that the advancement in age is often accompanied by an increasing affinity with earlier generations and a shift into a more cosmic and transcendent one [36]. The original family is usually the most important primary agent of socialization, a process through which an individual´s identity is formed through the act of interaction with others [1]. The informants were clearly proud of being able to maintain and develop this social heritage when aging in Sweden with their families. Having a social history provided them with a sense of continuity and coherence. The continuity theory of normal aging [37] claims that an individual has certain internal and external structures, such as personality, beliefs, relationships, and social roles, that remain constant throughout the course of life. It is just this continuity that enables older people, by using strategies tied to their past experiences of themselves and their social world, to maintain a strong sense of purpose and self in the face of coping with the challenges of aging [37].

There was some variation in the structure of the family the informants lived in. Although majority of them expressed a nostalgia about the warm loving big families they once grew up in, they themselves had been living in nuclear families during their time in Sweden with their children and partners, and now alone with their partners when their grown-up children had built their own nuclear families. Others lived as contented single because they had their children and grandchildren as their family. Most of them also took great pleasure in having friends. Only one man argued that he cared more about recognition from fellow people in the society than personal relations. What they all agreed on was the need of community and solidarity, and the need to have a role or roles in a social context. Together, they contributed to a broad societal perspective on family - family as the primary cell of society and society functioning as a big family, with community and solidarity between fellow human beings as well as between generations. This illustrates that it is not the structure but the function of the family – instrumental and emotional support to its members - that matters and endures. In one sense, the term family can be seen as a metaphorical expression of belongingness. A sense of belonging is a basic need of humans [38], and this need does not diminish as we age. Having a sense of belonging means that the older person is connected to others as part of a group, being able to participate, being accepted, recognized, and valued by others [39], just as one feels that one belongs to the family.

### Methodological reflections

The results of this study should be viewed with some limitations in mind. Despite the variation in the informants’ demographic, cultural, and socioeconomical backgrounds, these 15 informants may not be representative of this heterogenic target population. This study should be seen as a pilot study. Further studies are needed to pick up a wider range of experiences from older foreign-born who have lived less time in Sweden, or being childless, or living with more mobility impairment, who are not fairly represented in this study.

Our study is not free of the biases, assumptions, and the personality of the researchers. The fact that the first author and the interviewer being herself foreign-born living in Sweden and working clinically as a geriatric nurse might have impacted both data generation and data analysis. Remaining authors are also healthcare professionals with great experience of doing qualitative research, thus reflecting on how professional background, personal experiences and value orientation might affect the interpretation of the findings has continuously been a part of the research process.

A complementary ethics application was submitted and approved for use by interview interpreters with the aim of reaching out to the part of the target population that would be otherwise difficult to reach because of a language barrier. One relative with tested language ability was trusted as the interpreter for two participants. We are aware that relatives have an emotional involvement, which may have affected the information that the participants were willing to share. However, we still consider the benefit of this important contribute of knowledge should have outweighed the disadvantages.

Finally, it was our point of departure to employ grounded theory to generate theory with explanatory power, however, the study turns out to be more of an in-depth descriptive analysis of family relationships using grounded theory approach. Though we did not reach conceptualization abstract of time, place, and person [27], the grounded theory approach has contributed to deepening our understanding of the social context for aging by studying the interrelationship between the informants´ personal interpretation of family and the reality they lived in.

## Conclusion and implication for practice

The older foreign-born experienced life satisfaction as they aged with their families. Family meant community and solidarity. It was in the family that they found their different roles that had defined them. Family was an indispensable part of their social identity. The findings highlight the importance of older foreign-born being studied from a lifetime perspective and a family perspective.

## Data Availability

All the data generated, used, and analysed during the current study are stored on Linköping University´s secured server, and are accessible to the authors only. The datasets generated and/or analysed during the current study are not publicly available to protect the research informants’ privacy. However, these datasets are available from the corresponding author on reasonable request.
